# Autoantibodies as Precision Tools in Connective Tissue Diseases: From Epiphenomenon to Endophenotype

**DOI:** 10.3390/antib15010007

**Published:** 2026-01-13

**Authors:** Muhammad Soyfoo, Julie Sarrand

**Affiliations:** Department of Rheumatology, Hôpital Universitaire de Bruxelles (HUB), 1070 Bruxelles, Belgium; julie.sarrand@ulb.be

**Keywords:** autoantibodies, connective tissue disease, precision medicine, endotypes, autoimmunity

## Abstract

Autoantibodies have long been regarded as passive reflections of immune dysregulation in connective tissue diseases (CTDs). Recent advances in systems immunology and molecular pathology have fundamentally redefined them as active molecular fingerprints that delineate distinct disease endophenotypes with predictive power for clinical trajectories and therapeutic responses. Rather than mere epiphenomena, autoantibodies encode precise information about dominant immune pathways, organ tropism, and pathogenic mechanisms. This review synthesizes emerging evidence that autoantibody repertoires—defined by specificity, structural properties, and functional characteristics—stratify patients beyond traditional clinical taxonomy into discrete pathobiological subsets. Specific signatures such as anti-MDA5 in rapidly progressive interstitial lung disease, anti-RNA polymerase III in scleroderma renal crisis, and anti-Ro52/TRIM21 in systemic overlap syndromes illustrate how serological profiles predict outcomes with remarkable precision. Mechanistically, autoantibody pathogenicity is modulated by immunoglobulin isotype distribution, Fc glycosylation patterns, and tissue-specific receptor expression—variables that determine whether an antibody functions as a biomarker or pathogenic effector. The structural heterogeneity of autoantibodies, shaped by cytokine microenvironments and B-cell subset imprinting, creates a dynamic continuum between pro-inflammatory and regulatory states. The integration of serological, transcriptomic, and imaging data establishes a precision medicine framework: autoantibodies function simultaneously as disease classifiers and therapeutic guides. This endophenotype-driven approach is already influencing trial design and patient stratification in systemic lupus erythematosus, systemic sclerosis, and inflammatory myopathies, and is reshaping both clinical practice and scientific taxonomy in CTDs. Recognizing autoantibodies as endophenotypic determinants aligns disease classification with pathogenic mechanism and supports the transition towards immunologically informed therapeutic strategies.

## 1. Introduction: The Evolution of Autoantibody Biology

The history of autoimmunity is inseparable from the discovery of autoantibodies. Since Hargraves’ observation of lupus erythematosus cells in 1948 [1] and Coons’ development of immunofluorescence microscopy [2], circulating immunoglobulins targeting self-antigens have served as the most recognizable molecular signatures of connective tissue diseases (CTDs). For decades, their primary value resided in diagnosis: antinuclear antibodies (ANAs) as hallmarks of systemic lupus erythematosus (SLE) [3], anti-centromere antibodies marking limited systemic sclerosis (SSc) [4], and anti-Jo-1 heralding inflammatory myopathy [5]. For decades, the laboratory report functioned as a diagnostic talisman, even as autoantibodies were largely regarded as epiphenomena—mere by-products of immune dysregulation rather than mechanistic drivers of disease. This reductionist perception has undergone radical revision. The convergence of systems immunology, high-throughput proteomics, and clinical deep phenotyping has reframed autoantibodies as windows into the mechanistic architecture of systemic disease. Rather than sheer vague by-products of immune activation, autoantibodies are construed as structured outputs of selective immunological pathways that govern organ tropism, inform prognosis, and anticipate therapeutic response [6,7]. Current evidence indicates that each connective tissue disease is not a single entity but a constellation of antibody-defined endophenotypes—biologically distinct subsets that may share clinical features while diverging profoundly in underlying mechanisms [8,9].

### 1.1. From Epiphenomenon to Endophenotype

The concept of endophenotypes—intermediate biological states linking genotype to phenotype—originated in neuropsychiatry but has gained profound resonance in immunology [10]. In CTDs, autoantibodies function as natural reporters of aberrant immune circuits, encapsulating information about initiating triggers, the quality of T-B cell cooperation, and the cytokine milieu sustaining chronic inflammation [11]. The recurring association between specific autoantibodies and organ-specific damage illustrates their precision: anti-MDA5 predicts rapidly progressive interstitial lung disease (RP-ILD) in dermatomyositis with 70–90% positive predictive value [12]; anti-RNA polymerase III identifies scleroderma renal crisis risk and paraneoplastic disease [13]; anti-Ro (anti-SSA/TRIM21) signals systemic involvement and elevated lymphoma risk in Sjögren’s disease (SjD) [14]; anti-C1q correlates with lupus nephritis flares [15]. These relationships are not arbitrary—they reflect biologically coherent pathways where antigen expression, tissue accessibility, and local immune context intersect.

Mechanistic investigations increasingly support the functional role of autoantibodies beyond passive marking. They participate directly in tissue injury through immune complex formation [16], complement activation [17], and Fcγ receptor (FcγR) signaling [18]. Critically, the pro- or anti-inflammatory potential of an antibody depends on structural variables—isotype, IgG subclass, and Fc glycosylation pattern—that modulate affinity for activating or inhibitory receptors [19,20]. Cytokine analyses demonstrate that reduced fucosylation or incomplete terminal galactosylation markedly enhances inflammatory effector functions, establishing a direct link between antibody molecular structure and immunological activity [21,22]. It is important to distinguish between established mechanistic evidence and emerging hypotheses. While in vitro studies demonstrate that hypogalactosylated and afucosylated IgG enhance FcgammaRIIIa binding and ADCC, the causal relationship between glycosylation changes and disease activity in vivo remains largely associative. Longitudinal studies show that glycan profiles correlate with flares, but whether they drive pathology or merely reflect underlying B-cell dysregulation is unresolved. Similarly, the functional consequences of autoantibody glycosylation are inferred primarily from passive transfer models and in vitro assays rather than direct patient-derived evidence. Future studies employing glycoengineered monoclonal antibodies and genetic approaches targeting glycosyltransferases will be essential to establish causality.

### 1.2. The Organizational Canvas of Humoral Autoimmunity

Large-scale serological profiling has fundamentally reshaped our understanding of the organizational architecture underlying autoantibody networks in connective tissue diseases. In systemic lupus erythematosus (SLE), hierarchical clustering of autoantibody specificities reveals discrete modules corresponding to interferon-driven versus complement-deficient endophenotypes. In systemic sclerosis (SSc), the canonical triad of anti-centromere, anti-topoisomerase I (anti-Scl-70), and anti-RNA polymerase III antibodies defines largely mutually exclusive molecular clusters, each associated with distinct transcriptomic programs, vascular pathology, and organ involvement [23,24,25,26,27,28,29,30,31,32,33,34,35]. Similar stratification characterizes idiopathic inflammatory myopathies, where antibodies such as anti-TIF1γ, anti-MDA5, anti-HMGCR, and anti-Mi-2 delineate coherent clinical syndromes more accurately than traditional histopathology [36,37,38,39,40,41,42,43,44,45,46,47,48]. Collectively, these patterns indicate that autoantibodies function as durable readouts of pathogenic programs initiated by environmental triggers acting on genetically and epigenetically susceptible hosts. These observations highlight a central paradox of connective tissue diseases: an immune system capable of extraordinary antigenic precision simultaneously generates marked clinical heterogeneity. Autoantibodies act as molecular archives of this process, preserving evidence of antigenic exposure, B-cell lineage maturation, and the selective pressures imposed by cytokine milieu and T-cell help. Their evolving specificity often mirrors disease chronology; in Sjögren’s disease, for example, early anti-Ro and anti-La responses may precede diversification toward ribonucleoprotein targets, reflecting progressive epitope spreading over time. From a translational perspective, recognizing autoantibodies as endophenotypic determinants has major implications for diagnosis, prognosis, therapeutics, and clinical trial design. Serological stratification refines diagnostic precision in clinically overlapping syndromes, distinguishing, for instance, anti-PL-7/PL-12–associated antisynthetase phenotypes from classic polymyositis, which require fundamentally different therapeutic strategies. Prognostically, specific autoantibody profiles signal heightened risk for organ-threatening complications, including anti-MDA5 antibodies for rapidly progressive interstitial lung disease [40,41,42,43,44], anti-RNA polymerase III antibodies for scleroderma renal crisis and malignancy surveillance [49,50,51,52,53], and anti-Ro52 antibodies for increased lymphoma susceptibility [54,55,56,57,58,59,60,61,62,63,64].

Therapeutically, accumulating evidence suggests that serology may anticipate differential treatment responses, while emerging data indicate that IgG Fc glycosylation patterns may predict responsiveness to intravenous immunoglobulin. Incorporating antibody-based stratification into clinical trial frameworks reduces biological heterogeneity, sharpens endpoint sensitivity, and accelerates drug development, as illustrated by initiatives such as PRECISION-SLE and DECODE-SSc. Longitudinal analyses further show that effective B-cell–directed therapy remodels autoantibody networks, suggesting that serological “re-education” parallels clinical remission and may mark a transition from nonspecific immunosuppression toward immune tolerance within precision-medicine paradigms.

### 1.3. Conceptual Framework

If CTDs represent orchestral compositions of immune dysregulation, autoantibodies are not background noise—they are distinct instruments whose tone reveals the underlying score. This review explores the molecular genesis, structural diversity, and functional implications of autoantibody repertoires across major connective tissue diseases. We argue that understanding autoantibodies as endophenotypic determinants provides conceptual scaffolding for a new immunological taxonomy of rheumatic disease—one aligning classification with mechanism, and mechanism with therapeutic strategy. In this emerging landscape, the future of rheumatology will be shaped in the alphabet of autoantibodies.

## 2. Molecular Genesis of Autoantibody Repertoires

One of the most fascinating and yet dangerous processes lies in autobodies generating diversity within a system that is initially designed to discriminate self from non-self. In CTDs, this machinery—normally devoted to pathogen defense—becomes subtly misdirected, creating antibody repertoires targeting endogenous molecules with exquisite precision. Understanding autoantibody emergence requires revisiting B-cell tolerance architecture, the checkpoints that fail, and environmental forces driving chronic production.

### 2.1. B-Cell Tolerance: The Fragile Equilibrium

B cells develop in bone marrow under central tolerance surveillance. Immature clones expressing autoreactive receptors undergo clonal deletion, receptor editing, or anergy. Yet these checkpoints are inherently leaky; estimates suggest 30–50% of newly generated B cells display some autoreactivity. Peripheral tolerance mechanisms—follicular exclusion, inhibitory receptor signaling (FcγRIIB), and regulatory T and B cells—provide secondary safeguards. Breakdown at any stage permits self-reactive clones to mature, seed germinal centers, and undergo affinity maturation against self-antigens [11,12,13,65,66,67,68,69,70].

Genetic predispositions subtly tilt this equilibrium. Variants in *PTPN22, BANK1, BLK, and TNFAIP3* alter B-cell receptor (BCR) signaling thresholds, lowering activation barriers for self-antigens. Defects in complement components (C1q, C4) impair immune complex clearance, sustaining antigenic stimulation. The cumulative effect creates landscapes where autoreactive B cells persist in subclinical readiness, awaiting environmental signals for full activation.

### 2.2. Environmental Triggers and Molecular Mimicry

Transformation from silent autoreactivity to overt autoimmunity often follows environmental provocation. Viral infections provide classic examples [11,12,65]: Epstein–Barr virus (EBV), cytomegalovirus (CMV), and parvovirus B19 harbor epitopes mimicking autoantigens including Ro60, Sm, and topoisomerase I. Through molecular mimicry, these pathogens awaken quiescent autoreactive clones sustained via cross-reactive antigens. Chronic infection induces type I interferon (IFN-I) responses—cytokine environments favoring plasmablast differentiation and somatic hypermutation.

Beyond infection, tissue injury and oxidative stress generate neoantigens—self-proteins modified by carbamylation, citrullination, or oxidation. These post-translational modifications alter epitope conformation, creating “altered self” escaping immune tolerance. In rheumatoid arthritis, citrullination of vimentin and enolase initiates anti-citrullinated protein antibody (ACPA) production years before clinical disease. Similar processes operate in CTDs: apoptotic blebs exposing Ro and La antigens in Sjögren’s disease, or DNA-topoisomerase complexes externalized during cell stress in SSc. Immunogenic debris feeds cycles of epitope exposure, presentation, and affinity maturation.

### 2.3. The Innate Bridge: Interferon and Nucleic Acid Sensing

Innate immunity provides ignition signals for sustained autoantibody formation. Endosomal sensors (TLR7, TLR9) recognize RNA- and DNA-containing immune complexes [71,72,73,74,75,76,77,78,79,80,81,82,83], triggering plasmacytoid dendritic cell (pDC) activation and massive IFN-I release. This “interferon signature,” pervasive across SLE, SSc, and myositis, lowers B-cell activation thresholds, promotes class-switch recombination, and drives BAFF (B-cell activating factor) expression. Elevated BAFF extends B-cell survival while IFN-induced transcriptional programs stabilize autoreactive plasma cells within inflamed tissue niches.

Synergy between autoantibody and interferon pathways creates self-sustaining feedback loops [71,72,73,74,75,76,77,78,79,80]. Autoantibodies form immune complexes delivering self-nucleic acids to endosomal sensors; resulting interferon bursts amplify antibody production. This cycle blurs distinctions between cause and consequence—illustrating how “epiphenomena” become disease perpetuators.

### 2.4. Germinal Centers and Autoantibody Diversification

Once tolerance breaches occur, germinal centers (GCs) become crucibles of autoimmune maturation. Within these microanatomical structures, B cells undergo somatic hypermutation (SHM) and class-switch recombination (CSR) [11,13,65,68] under influence of T follicular helper (Tfh) cells and activation-induced cytidine deaminase (AID). In physiological immunity, these processes refine affinity for foreign antigens; in CTDs, they diversify autoreactivity.

Chronic immune activation creates ectopic germinal centers within target tissues [56,58,62,63,64]—salivary glands in SjD, synovium in rheumatoid arthritis, muscle in myositis, lung in SSc. These tertiary lymphoid structures (TLS) perpetuate antigen-driven selection in situ, enabling continuous local antibody repertoire evolution. Transcriptomic analyses reveal clonal B-cell expansions expressing VH gene segments biased towards autoreactivity and enriched in IgG1/IgG3 subclasses—potent complement and FcγR activators. TLS persistence explains chronicity and organ specificity of autoantibody production long after systemic inflammation wanes.

### 2.5. Epitope Spreading: The March of Specificity

A striking feature of autoantibody evolution is epitope spreading [19,84,85]—immune responses diversifying from single initiating epitopes to multiple epitopes within the same or related antigens. This occurs through intermolecular help between B and T cells recognizing distinct but physically associated antigens. In lupus, early anti-Ro responses lead to anti-La and anti-Sm; in SSc, anti-topoisomerase I antibodies diversify toward other nucleic acid-binding proteins. Progressive epitope target expansion transforms initially focused autoimmune responses into polyclonal serological signatures mirroring disease progression.

Temporal mapping of preclinical cohort sera demonstrates autoantibody diversification precedes overt symptoms by years [19,86], suggesting immune systems “rehearse” disease long before clinical presentation. This has profound implications for early diagnosis and preventive intervention strategies.

### 2.6. B-Cell Subsets and Plasmablast Dynamics

Modern cytometry and single-cell RNA sequencing delineate distinct B-cell subsets implicated in autoantibody production. Age-associated B cells (ABCs), characterized by T-bet and CD11c expression, accumulate in autoimmunity [11,13] and display heightened TLR7 responsiveness. They act as precursors to autoreactive plasma cells, correlating with disease flares in SLE and SSc. Transitional B cells, normally tolerogenic, also display altered signaling profiles in CTDs, potentially seeding autoreactive pools.

Peripherally, short-lived plasmablasts dominate during flares while long-lived plasma cells—residing in bone marrow and inflamed tissues—maintain chronic seropositivity. These long-lived cells resist conventional B-cell depleting therapies like rituximab [87,88,89,90,91,92,93,94,95,96,97,98,99,100,101,102], explaining autoantibody titer persistence despite clinical remission. Therapeutic strategies now explore targeting survival niches (CXCL12-CXCR4 axis) or plasma cell metabolic dependencies for deeper immunologic control.

### 2.7. Genetic and Epigenetic Imprinting

Genetic backgrounds dictate not only susceptibility but also autoantibody specificity. HLA class II alleles (DRB103:01, 15:01, 04:01) shape peptide presentation [8,9,50,60], determining which self-epitopes breach tolerance. Genome-wide association studies reveal distinct risk architectures: anti-MDA5-positive myositis associates with HLA-DRB104:05 in East Asian populations; anti-RNA polymerase III positivity in SSc links to *POLR3A* gene polymorphisms.

Epigenetic mechanisms—DNA methylation, histone acetylation, and non-coding RNAs [61,83]—further influence B-cell differentiation and antibody production. Hypomethylation of interferon-regulated genes primes B cells for hyperresponsiveness; microRNAs like miR-155 modulate class switching and plasma cell differentiation. These molecular layers combine to produce distinctive, stable yet adaptable autoantibody repertoires characterizing CTDs.

### 2.8. Structural Evolution: Glycosylation and Affinity Maturation

Autoantibody function extends beyond specificity; structural modifications profoundly affect pathogenic potential. IgG glycosylation patterns, modulated by inflammatory cytokines and metabolic states [103,104,105,106,107,108,109,110,111,112,113], determine FcγR engagement and complement activation. Hypogalactosylated IgG, common in active rheumatoid arthritis and SLE, displays increased pro-inflammatory potential. IgA autoantibodies—often overlooked—play roles in mucosal immunity and systemic inflammation, with altered glycosylation contributing to immune complex deposition in vasculature and kidneys.

Somatic mutations within variable regions further refine affinity and may create public clonotypes—shared antibody sequences across unrelated patients. Such convergence implies selective pressure toward common autoantigenic motifs, evidencing that autoimmunity follows evolutionary logic rather than random chaos.

### 2.9. Synthesis: A Dynamic Immunological Ecosystem

Collectively, these processes—genetic predisposition, environmental insult, innate amplification, and adaptive diversification—forge complex, evolving autoantibody networks characterizing CTDs. Far from static markers, autoantibody repertoires constitute dynamic ecosystems fluctuating with disease phase, therapy, and tissue microenvironment. The shift from linear causation to network thinking is crucial: autoantibodies emerge not from single broken mechanisms but from distributed systems where tolerance, repair, and inflammation coexist in unstable equilibrium.

## 3. Structural Modifiers of Autoantibody Function

If specificity defines where autoantibodies act, structure determines how they behave. The same epitope can be recognized by antibodies with opposite immunological consequences—pro-inflammatory or regulatory—depending on Fc domain architecture, attached glycans, and isotype context. In CTDs, structural subtleties dictate whether autoantibodies quietly report immune dysregulation or ignite tissue damage.

### 3.1. The Fc Region: Commanding Immune Responses

The Fab fragment provides exquisite antigen recognition, but the Fc portion commands the immune orchestra. Through it, antibodies engage complement [114,115,116,117], FcγRs, and neonatal Fc receptor (FcRn) pathways controlling half-life and distribution.

#### 3.1.1. Complement Activation

Complement activation is primarily mediated by IgG1 and IgG3 subclasses [118,119,120,121,122,123,124,125,126], whose Fc domains bind C1q initiating the classical cascade. This mechanism underlies glomerular injury in lupus and vascular damage in antiphospholipid syndrome. Conversely, IgG4—dominant in certain anti-centromere or anti-DFS70 responses—is poor at complement fixation and often correlates with indolent or protective phenotypes.

#### 3.1.2. FcγR Engagement

FcγR engagement provides a second variability axis. Balance between activating (FcγRIIA, FcγRIIIA) and inhibitory (FcγRIIB) receptor signaling [119,127,128,129,130,131] determines whether immune complexes provoke inflammation or tolerance. In SLE and SSc, genetic polymorphisms lowering FcγRIIB expression or increasing FcγRIIA affinity for IgG1/3 amplify inflammation, explaining variable disease severity in patients with identical autoantibody specificities.

#### 3.1.3. FcRn Recycling

FcRn binding recycles IgG, extending antibody half-life from days to weeks and shaping serum titers. Novel therapeutics (efgartigimod, rozanolixizumab) exploit FcRn blockade [132,133,134,135,136,137,138,139,140,141,142,143,144,145,146] to accelerate IgG degradation, rapidly lowering pathogenic antibody levels. Clinical success of these agents in refractory lupus, myositis, and pemphigus proves that structural manipulation of antibody lifespan translates into therapeutic benefit.

### 3.2. Glycosylation: The Functional Tuning Dial

**Figure 1:**
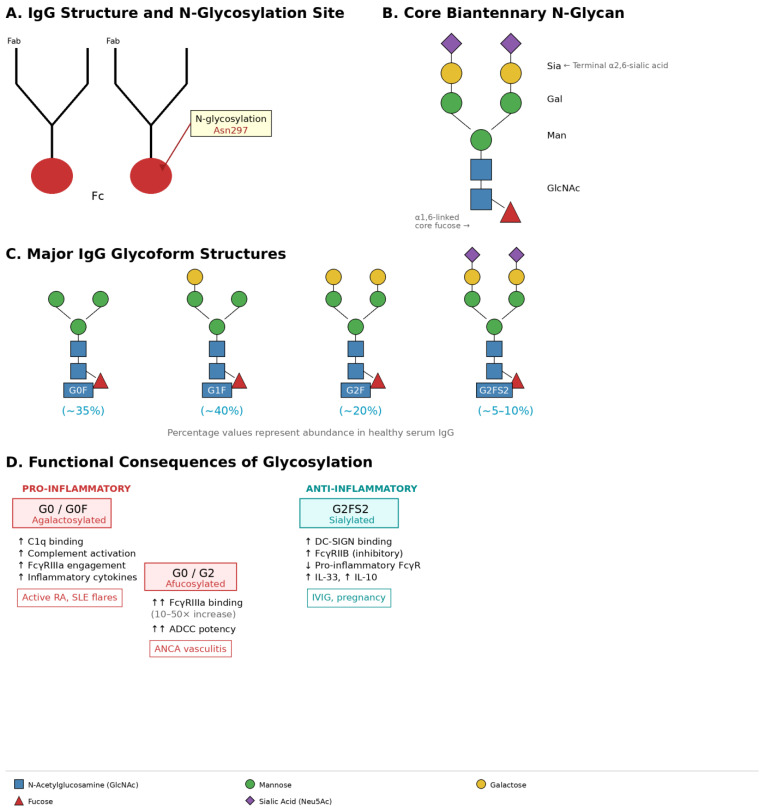
IgG Fc Glycosylation: Structural Diversity and Functional Consequences. (**A**) Schematic representation of IgG structure highlighting the Fc region and N-glycosylation sites at Asn297. (**B**) Core biantennary N-glycan structure showing the conserved heptasaccharide core with potential modifications. (**C**) Major IgG glycoform structures (G0F, G1F, G2F, G2FS2) with their relative abundance in healthy serum. (**D**) Functional consequences of glycosylation patterns: agalactosylated forms (G0F) promote complement activation and inflammatory FcγR engagement; afucosylated forms dramatically enhance FcγRIIIA binding and ADCC; sialylated forms (G2FS2) engage DC-SIGN and promote anti-inflammatory responses. Color coding: blue = GlcNAc, green = mannose, orange = galactose, red = fucose, purple = sialic acid.

The conserved N-glycosylation site at Asn297 in the CH2 domain of IgG represents one of the most consequential post-translational modifications in immunology. This single asparagine-linked biantennary glycan—occupying the interdomain space between the two CH2 regions—acts as a molecular rheostat that fine-tunes antibody effector function without altering amino acid sequence or antigen specificity. Far from being a static decoration, the Fc glycan undergoes dynamic remodeling in response to inflammatory signals, directly translating immune activation status into functional consequences (Figure 1).

#### 3.2.1. Structural Glycoforms and Their Functional Consequences

IgG Fc glycans exist as heterogeneous mixtures of related structures built on a conserved heptasaccharide core: two N-acetylglucosamines (GlcNAc) attached to Asn297, branching to three mannose residues, which extend into two antennae each terminating in additional GlcNAc. This core can be further modified by four key enzymatic additions, each conferring distinct functional properties:

Core Fucosylation: α1,6-linked fucose attached to the innermost GlcNAc is the most common modification, present on >90% of serum IgG in healthy individuals. Core fucose sits in the space between CH2 domains, sterically hindering FcγRIIIA engagement. Removal of this single monosaccharide—generating “afucosylated” IgG—dramatically enhances FcγRIIIA binding affinity [147,148] (10–50-fold increase) and amplifies ADCC potency, a property now exploited therapeutically in glycoengineered monoclonal antibodies (obinutuzumab, mogamulizumab).

Galactosylation: Terminal β1,4-linked galactose can be added to one (G1) or both (G2) antennae, or absent entirely (G0). Galactose levels correlate inversely with inflammatory potential: G0 structures lacking terminal galactose expose terminal GlcNAc residues that promote C1q binding and complement activation [103,107,108,109,110]. The penultimate GlcNAc on agalactosylated structures also interacts with mannose-binding lectin (MBL), activating the lectin complement pathway. Conversely, galactosylated IgG (G1/G2) exhibits reduced complement fixation and attenuated inflammatory capacity.

Sialylation: α2,6-linked sialic acid (N-acetylneuraminic acid, Neu5Ac) represents the terminal modification on galactosylated structures. Despite comprising only ~10% of serum IgG in healthy individuals, sialylated IgG exerts potent anti-inflammatory effects through multiple mechanisms. Sialic acid redirects antibody-receptor interactions from activating FcγRs toward inhibitory pathways: sialylated Fc preferentially binds DC-SIGN and SIGN-R1 on dendritic cells and macrophages [104,105,149], triggering IL-33 release and upregulating FcγRIIB expression on effector cells. This creates a negative feedback loop dampening immune activation. Intravenous immunoglobulin (IVIG)—used therapeutically for its anti-inflammatory properties—derives much of its efficacy from its enriched sialylated fraction (up to 25% in some preparations).

Bisecting GlcNAc: A fifth GlcNAc can be β1,4-linked to the core mannose by GnT-III (MGAT3), creating a “bisecting” structure that prevents further antenna elongation. Bisecting GlcNAc correlates with enhanced ADCC [109,131,150] through mechanisms not fully elucidated, though structural studies suggest altered CH2 domain positioning that facilitates receptor engagement.

These modifications combine to generate at least 36 distinct glycoforms in human serum IgG. The most abundant structures can be abbreviated using standardized nomenclature:

- G0F: Agalactosylated, fucosylated (most abundant in healthy individuals, ~35%);
- G1F: Mono-galactosylated, fucosylated (~40%);
- G2F: Di-galactosylated, fucosylated (~20%);
- G2FS2: Di-galactosylated, fucosylated, di-sialylated (~5–10%);
- G0: Agalactosylated, afucosylated (<5%).

The relative abundance of these glycoforms determines the net inflammatory potential of the antibody repertoire.

#### 3.2.2. Inflammatory Regulation of Glycosylation Machinery

The glycan profile of IgG is not genetically fixed but rather responds dynamically to inflammatory signals through coordinated regulation of glycosyltransferases and glycosidases in plasma cells and plasmablasts. The “glycan switch” observed during immune activation reflects transcriptional and post-transcriptional control of the glycosylation machinery.

Galactosyltransferases: β1,4-galactosyltransferase 1 (B4GALT1) adds terminal galactose to GlcNAc-terminating structures. During acute inflammation, B4GALT1 expression is suppressed in activated B cells through multiple pathways. Pro-inflammatory cytokines (TNF-α, IL-6, IL-1β) activate NF-κB [107,108,110], which competes with transcription factors required for B4GALT1 expression. Type I interferons—elevated in SLE and other autoimmune conditions—directly repress B4GALT1 transcription through STAT1-mediated mechanisms. Consequently, plasmablasts generated during inflammatory responses produce predominantly agalactosylated (G0) antibodies. This effect is remarkably rapid: B4GALT1 downregulation begins within 24–48 h of inflammatory stimulus and recovers weeks to months after resolution.

The metabolic state of plasma cells also influences galactosylation. B4GALT1 activity requires UDP-galactose as substrate, generated through the Leloir pathway of galactose metabolism. Inflammatory activation shifts cellular metabolism toward glycolysis and away from oxidative phosphorylation, reducing UDP-galactose availability. Additionally, the acidic pH of inflammatory microenvironments (pH 6.5–7.0) reduces B4GALT1 enzymatic activity, which peaks at physiological pH 7.4.

Sialyltransferases: ST6GAL1 (β-galactoside α2,6-sialyltransferase 1) adds terminal sialic acid [104,105,110,111] to galactosylated structures. ST6GAL1 expression is tightly regulated by inflammation: IL-6 suppresses ST6GAL1 transcription via STAT3-mediated mechanisms, while IL-10 (anti-inflammatory) enhances expression. The substrate for ST6GAL1—CMP-sialic acid—is also limiting during inflammation due to increased sialic acid catabolism and reduced biosynthesis. Consequently, inflammatory conditions produce a double deficit: reduced galactosylation provides fewer substrates for sialylation, and decreased ST6GAL1 activity further limits sialylation of available galactosylated structures.

Regulatory T cells (Tregs) secrete IL-10 and TGF-β, which upregulate ST6GAL1 in plasma cells, promoting production of anti-inflammatory sialylated IgG. This represents a homeostatic mechanism to limit inflammation. In autoimmune diseases with Treg dysfunction (SLE, RA), this brake on inflammation is impaired, perpetuating production of pro-inflammatory hypogalactosylated IgG.

Fucosyltransferases: FUT8 (α1,6-fucosyltransferase) adds core fucose and is constitutively active in most plasma cells. However, certain activation states—particularly in germinal centers with strong CD40L/CD40 signaling—can suppress FUT8, generating afucosylated antibodies with enhanced ADCC capacity. This pathway may be exploited during certain infections but appears dysregulated in some autoimmune contexts where afucosylated autoantibodies amplify tissue damage.

GnT-III (MGAT3): The enzyme adding bisecting GlcNAc is upregulated by IL-6 through unclear mechanisms. Bisecting structures are more prevalent in inflammatory conditions, though their functional significance in autoimmunity remains incompletely understood.

#### 3.2.3. Disease-Specific Glycosylation Signatures

Glycomic profiling has revealed disease-specific signatures that correlate with pathogenesis and may serve as biomarkers or therapeutic targets.

Rheumatoid Arthritis (RA):

RA exhibits the most dramatic and best-characterized glycan switch. During active disease, IgG—particularly anti-citrullinated protein antibodies (ACPA) and rheumatoid factor—shows profound agalactosylation, with G0 structures comprising 50–60% of total IgG (versus 35% in healthy controls). This shift precedes clinical symptoms: agalactosylated IgG can be detected months to years before RA onset [107,108], suggesting a pathogenic role rather than merely reflecting inflammation.

The functional consequences are substantial: agalactosylated ACPA forms larger immune complexes, activates complement more efficiently, and binds FcγRs with altered specificity, favoring activating receptors. Synovial tissue in RA contains plasma cells with markedly suppressed B4GALT1 expression, producing locally agalactosylated antibodies that perpetuate joint inflammation.

Therapeutic interventions that induce remission (methotrexate, TNF inhibitors, IL-6 blockade) [91,92,110] partially restore galactosylation over 3–6 months. Patients who achieve sustained remission show near-normalization of galactose levels, while those with refractory disease maintain high G0 levels. This makes IgG galactosylation a potential marker of treatment response.

Intriguingly, pregnancy—which typically ameliorates RA—is associated with increased IgG galactosylation [107,108] driven by estrogen-mediated upregulation of B4GALT1. Disease flare post-partum coincides with return to agalactosylated profiles, mechanistically linking hormonal regulation of glycosylation to disease activity.

Systemic Lupus Erythematosus (SLE):

SLE shows a more complex glycosylation pattern reflecting disease heterogeneity. Active SLE features:

- Elevated G0 structures (45–50% vs. 35% in controls);
- Markedly reduced sialylation (3–5% vs. 10% in controls);
- Variable fucosylation depending on organ involvement.

The loss of sialylation is particularly pronounced and correlates strongly with disease activity (SLEDAI scores), complement consumption [108,109,151] (low C3/C4), and anti-dsDNA titers. Mechanistically, reduced sialylation impairs the IVIG-like tolerogenic effects that sialylated IgG normally provides, removing a homeostatic brake on inflammation.

SLE IgG also exhibits altered antenna branching and increased bisecting GlcNAc, features associated with enhanced complement activation. This may explain why SLE immune complexes are particularly potent complement activators despite not always showing the most extreme agalactosylation.

Organ-specific patterns emerge: lupus nephritis patients show more severe galactose loss than those with primarily cutaneous disease. Neuropsychiatric lupus correlates with specific glycan signatures including increased core-fucosylation and reduced sialylation of IgG in cerebrospinal fluid.

Anti-malarial drugs (hydroxychloroquine)—which alkalinize endosomal pH and may affect glycosylation machinery—partially restore galactosylation in some SLE patients [108,110], potentially contributing to their therapeutic efficacy beyond TLR inhibition.

ANCA-Associated Vasculitis (AAV):

AAV presents a distinct glycosylation profile characterized by:

- Moderate agalactosylation (40–45% G0);
- Markedly reduced fucosylation (15–20% afucosylated vs. <5% in controls);
- Near-complete loss of sialylation (<2%).

The loss of core fucose on ANCA (anti-MPO and anti-PR3 antibodies) dramatically enhances their pathogenicity. Afucosylated ANCA binds FcγRIIIA on neutrophils with 20–50-fold higher affinity [108,109,147,148], potently triggering degranulation, respiratory burst, and NETosis even at low antibody titers. This explains why some patients with low ANCA levels by ELISA still develop severe vasculitis—the glycosylation status determines functional potency independent of total antibody concentration.

Longitudinally, afucosylated ANCA levels correlate with disease activity and predict relapse. Patients in remission show partial recovery of fucosylation (though rarely to normal levels), and re-emergence of highly afucosylated ANCA precedes clinical relapse by weeks to months.

The mechanisms driving loss of fucosylation in AAV remain incompletely understood but may involve chronic interferon-γ signaling (which suppresses FUT8) or intrinsic abnormalities in B cell glycosylation machinery. Notably, the FUT8 gene locus shows rare variants associated with AAV susceptibility in some populations.

Sjögren’s Disease (SjD):

Glycosylation in SjD shows intermediate features:

- Moderately elevated G0 (40–42%);
- Reduced but not absent sialylation (6–8%);
- Normal fucosylation;
- Increased bisecting GlcNAc.

The glycan profile correlates with systemic disease activity (higher Focus Score, extraglandular manifestations) but not with sicca symptoms per se. Patients who develop lymphoma show progressive loss of glycan diversity months to years before malignant transformation, suggesting glycosylation monitoring might identify high-risk individuals.

Systemic Sclerosis (SSc):

SSc exhibits relatively preserved galactosylation compared to RA and SLE (38–40% G0), but shows unique features:

- Increased bisecting GlcNAc (particularly in diffuse cutaneous SSc);
- Reduced sialylation correlating with interstitial lung disease severity;
- Normal fucosylation in most patients.

The limited shift toward agalactosylation aligns with SSc’s progressive rather than relapsing-remitting course—suggesting less acute inflammatory activation of plasma cells. However, the increase in bisecting structures may contribute to the fibrotic phenotype through unclear mechanisms possibly involving enhanced TGF-β signaling.

#### 3.2.4. Therapeutic Implications and Future Directions

Understanding glycosylation opens multiple therapeutic avenues:

Glycan-Based Biomarkers: IgG galactosylation and sialylation levels may serve as biomarkers for disease activity, treatment response, and relapse prediction. Commercial assays using HILIC-UPLC or mass spectrometry can quantify glycoforms with clinical turnaround times of days. Afucosylated ANCA levels may refine ANCA testing to identify patients at highest risk for active vasculitis.

Critical appraisal of biomarker assays reveals significant challenges. IgG glycosylation profiling by mass spectrometry shows inter-laboratory coefficients of variation (CV) of 15–25% for minor glycoforms, limiting clinical utility. ELISA-based autoantibody quantification demonstrates better reproducibility (CV 8–15%) but lacks standardization across manufacturers; anti-MDA5 titers from different platforms are not directly comparable. Multiplexed bead arrays offer efficiency but suffer from matrix effects and cross-reactivity. Standardization efforts including WHO reference materials for anti-dsDNA have improved comparability, but most disease-specific autoantibodies lack international standards. Clinical feasibility is constrained by cost (USD 200–500 per comprehensive glycomic profile), turnaround time (3–7 days for specialized assays), and limited availability outside academic centers. Point-of-care testing remains elusive. These limitations must temper enthusiasm for biomarker-guided management until robust, standardized, and accessible assays are validated in prospective clinical trials.

Metabolic Interventions: Strategies to restore “healthy” glycosylation patterns are being explored:

- Galactose supplementation: Oral galactose increases UDP-galactose availability and modestly improves IgG galactosylation in some studies, though clinical efficacy remains unproven.
- Sialic acid precursors: N-acetylmannosamine increases CMP-sialic acid pools and enhances IgG sialylation in vitro; human trials are ongoing.
- Anti-inflammatory therapy timing: Early aggressive treatment may prevent the shift to pro-inflammatory glycosylation patterns, improving long-term outcomes.

Glycoengineered Therapeutics: The success of afucosylated anti-CD20 antibodies in hematologic malignancies demonstrates the therapeutic potential of glycan modification. Future autoimmune therapies might involve:

- Hypersialylated IVIG preparations with enhanced anti-inflammatory activity;
- Glycan-modified biologics optimized for specific Fc-FcγR interactions;
- Small molecule modulators of glycosyltransferases to correct aberrant glycosylation.

Targeting Glycosylation Machinery: Drugs that restore normal glycosyltransferase expression or activity could correct the glycan switch at its source. ST6GAL1 activators are in preclinical development, aiming to promote anti-inflammatory sialylated IgG production in situ.

The “glycan switch” represents a targetable mechanism linking inflammation to antibody function. As glycomic technologies advance, personalized medicine approaches may emerge where glycosylation profiling guides therapy selection—treating patients with predominantly afucosylated pathogenic antibodies differently from those with normal glycosylation who may have different underlying mechanisms. The intersection of glycobiology and immunology continues to yield insights with direct translational potential.

### 3.3. Isotype Distribution: Reading Immunological Landscapes

Class-switch recombination (CSR) tailors antibody constant regions to distinct effector niches. In CTDs, isotype distribution reveals immunological disease habitats.

#### 3.3.1. IgG Subclasses

IgG remains the canonical systemic effector, with four subclasses displaying distinct properties [106,118,128]. IgG1/3 activate complement and are inflammatory; IgG4 is non-inflammatory, often protective. The same target antibody can switch subtypes, changing disease phenotype—anti-Ro52 in healthy carriers is usually IgG4; during active disease it shifts to IgG1, same target but opposite effects.

#### 3.3.2. IgA: The Mucosal Signature

IgA autoantibodies, long overlooked, are increasingly recognized as mucosal origin markers. Elevated IgA anti-Ro and anti-CCP titers imply initial tolerance breaches may occur at mucosal surfaces [106,107] (oral, bronchial, intestinal), consistent with “mucosal autoimmunity” hypotheses. IgA’s ability to form polymeric immune complexes engaging FcαR (CD89) on neutrophils drives tissue-specific inflammation, particularly in the lung and kidney. Finding IgA autoantibodies suggests disease began at barrier surfaces, supporting environmental trigger roles.

#### 3.3.3. IgM: The Paradox

Natural IgM contributes to apoptotic cell clearance and may be protective, whereas somatically mutated IgM—as in cold agglutinin disease or cryoglobulinemia—becomes pathogenic. The balance influences vascular and renal outcomes in lupus and mixed cryoglobulinemic syndromes.

#### 3.3.4. Cytokine Control

Class-switch bias mirrors cytokine milieu: IFN-γ promotes IgG1/3, IL-4 favors IgG4, TGF-β induces IgA. Thus, isotype composition provides indirect readouts of inflammatory environments shaping disease, with IgG1:IgG4 ratios revealing dominant pathogenic pathways.

### 3.4. Immune Complexes: Pathogenic Scaffolds

Autoantibodies rarely act alone; they form immune complexes with target antigens that deposit in tissues or circulate to activate complement and FcRs. Complex size, charge, and clearance rates determine pathogenic potential.

In lupus nephritis, nucleosome-containing immune complexes lodge within glomerular basement membranes [75,76,85,152,153], initiating complement activation and neutrophil infiltration. In SSc, anti-endothelial cell immune complexes trigger oxidative stress and endothelial apoptosis, setting vasculopathy stages. In antiphospholipid syndrome, β2-glycoprotein I-containing complexes activate platelets and monocytes, transforming serological phenomena into thrombosis.

Modern imaging techniques (confocal microscopy, cryo-EM) reveal immune complexes act as molecular scaffolds, bringing together FcRs, complement receptors, and pattern-recognition sensors to form inflammatory “signalosomes.” Their spatial organization dictates downstream cytokine profiles—an architectural dimension of autoantibody pathogenicity previously underappreciated.

### 3.5. Tissue Tropism: Glycan-Receptor Crosstalk

Not all tissues respond equally to immune complexes. Local FcγR subset expression and complement regulators shape susceptibility to antibody-mediated injury. Glomerular endothelial cells express high FcγRIIA and C1q receptor levels [120,121,122,123,124,125,126,128,152,153], predisposing kidneys to immune complex deposition; pulmonary microvasculature enriched in FcγRIIIB on neutrophils explains anti-MDA5 and anti-Ro52 antibody predilection for lungs.

Glycan-dependent interactions further refine selectivity. Sialylated antibodies preferentially engage DC-SIGN-positive macrophages in liver and lymph nodes, eliciting tolerogenic responses, whereas afucosylated antibodies recruit cytotoxic NK cells within inflamed tissues. The “glycotope” of antibodies thus collaborates with receptor landscapes to dictate organ tropism—a concept with therapeutic implications for targeting specific effector axes.

### 3.6. Post-Translational Modifications Beyond Glycosylation

Other biochemical modifications subtly adjust antibody behavior. Oxidation and nitration of Fc methionine residues, common in chronic oxidative stress (SSc), decrease FcRn binding and accelerate catabolism, potentially explaining fluctuating titers. Carbamylation of antibody lysines, linked to uremic states, enhances complement activation. Succinylation and acetylation of Fab residues can alter antigen affinity, introducing microheterogeneity modulating pathogenicity over time.

Such modifications create an “antibody epigenome”—reversible chemical marks fine-tuning effector potential, analogous to histone modifications regulating gene expression.

### 3.7. B-Cell Subset Imprinting

Antibody structural identity is ultimately imprinted by producing B-cell subsets. Age-associated B cells and T-bet-positive plasmablasts secrete highly afucosylated [11,13,108], IgG1-biased antibodies under IFN-γ influence. Conversely, IL-10-producing regulatory B cells (Bregs) generate sialylated, less inflammatory IgG4. Cytokine gradients within TLS sculpt antibody glycoforms locally, explaining intra-tissue differences between serum and lesional antibodies in SLE, Sjögren’s syndrome, and myositis.

This dynamic implies therapeutic modulation of cytokine microenvironments—through JAK inhibition, interferon blockade, or BAFF/APRIL targeting—can reshape antibody quality even when total titers remain unchanged.

### 3.8. The Pathogenic Continuum

Traditional thinking divides antibodies into “pathogenic” and “benign.” Modern evidence favors a continuum where the same specificity oscillates between pathogenic and regulatory states depending on structure and context. Anti-Ro52 antibodies in healthy carriers are often IgG4-dominant and sialylated, exerting minimal effector activity; during systemic inflammation they shift toward IgG1/3, hypogalactosylated forms promoting FcγR engagement and cytokine release.

Similarly, natural IgM anti-phosphorylcholine antibodies protect against atherosclerosis, while class-switched IgG antiphospholipid antibodies induce thrombosis. Autoantibody function is therefore conditional—a product of molecular tuning rather than inherent malice.

### 3.9. Therapeutic Exploitation

Recognition of these modifiers has ushered in structure-targeted therapies:

- FcRn antagonists (efgartigimod, rozanolixizumab) reduce pathogenic IgG loads [132,133,134,135,136,137,138,139,140,141,142,143,144,145,146] in refractory lupus and myositis.
- Glycoengineering of therapeutic antibodies enables anti-inflammatory variants with enhanced sialylation.
- B-cell directed agents (ianalumab, belimumab) [89,90,102,154,155,156,157] indirectly reshape antibody glycosylation by modulating cytokine milieu.
- Experimental strategies aim to re-educate plasma cells producing tolerogenic isotypes—“antibody rehabilitation” rather than eradication.

### 3.10. Integrative Perspective

Structural and biochemical heterogeneity transforms autoantibodies from static serological markers into dynamic effectors capable of both injury and regulation. Pathogenicity arises not merely from self-recognition but from effector function miscalibration—excessive complement, insufficient inhibition, inappropriate glycan signaling, or tissue-specific receptor overexpression.

Understanding these modifiers offers dual benefits: mechanistic insight into disease pathogenesis and tangible therapeutic design targets. The next horizon is quantitative—integrating glycomics, subclass profiling, and FcR transcriptomics into composite “antibody functional scores” predicting outcomes more accurately than titer alone.

## 4. Disease-Specific Endophenotypes

The notion that autoantibodies are mere serological footprints is untenable. Across CTDs, discrete autoantibody repertoires define stable biological subsets differing in gene expression, organ involvement, and therapeutic response. Each autoantibody effectively “names” an immune endophenotype—a coherent pathobiology pattern expressed through immunity’s language of specificity.

### 4.1. Systemic Lupus Erythematosus: The Immune Mosaic

SLE harbors one of the most extensive and evolutionarily diverse autoantibody repertoires known. Traditional serology divides lupus into “nephritic,” “neuropsychiatric,” and “vascular” variants; molecular immunology reframes these as antibody-defined endophenotypes.

#### 4.1.1. Anti-dsDNA: The Nephritic Signature

Anti-dsDNA antibodies are emblematic but not monolithic. High-affinity, complement-fixing IgG1/3 forms correlate tightly with renal activity [14,15,22,152,153,158,159,160] (sensitivity 60–70% for active nephritis, specificity 95%) and complement consumption, while low-avidity or IgA isotypes may mark mucosal or vascular pathology. Their capacity to form nucleic acid-containing immune complexes links directly to pDC activation and interferon signatures. Transcriptomic clustering shows anti-dsDNA-positive individuals display IFN-regulated gene enrichment, whereas seronegative patients cluster within neutrophil-dominated or metabolic modules—distinct immune architectures under the same clinical label.

#### 4.1.2. Anti-C1q: The Complement-Intense Subset

Anti-C1q antibodies, frequently co-expressed with anti-dsDNA, amplify complement activation and renal immune complex deposition [75,120,121,122,123,124,125,126], defining a “complement-intense nephritic” subset with increased renal flare risk (hazard ratio 2.5–3.5).

#### 4.1.3. Antiphospholipid Antibodies: The Thrombotic Endophenotype

In contrast, antiphospholipid antibodies (aPL) delineate thrombo-inflammatory endophenotypes independent of classical lupus activity indices. aPL-positive SLE patients show tissue factor up-regulation and neutrophil extracellular trap formation [17,76], bridging humoral immunity with thrombosis biology.

#### 4.1.4. Structural Heterogeneity

Further nuance arises from autoantibody glycosylation: hypogalactosylated IgG promotes FcγR engagement and inflammation [103,104,105,106,107,108,109,110,111,118,127], while sialylated IgG exerts anti-inflammatory properties—illustrating that even within specificities, structure modulates phenotype.

#### 4.1.5. Therapeutic Implications

The SLE landscape reveals “lupus” as a federation of overlapping antibody-driven programs rather than a single disease. Integrating serological profiles with transcriptomic data redefines prognosis and suggests therapeutic matching: interferon blockade for IFN-high/anti-nucleic acid subsets [72,73,74,75,76,77,78,79,80,81,82,83,154,155,161,162], complement inhibition for anti-C1q nephritis [120,121,122,123,124,125,126], anticoagulation or NETosis modulation for aPL-mediated vasculopathy.

### 4.2. Systemic Sclerosis: Molecular Cartography

Few autoimmune diseases demonstrate more precise links between antibody specificity and clinical behavior than SSc. Here, serological profiles serve as molecular maps predicting fibrosis topography, vasculopathy, and malignancy risk.

#### 4.2.1. Anti-Centromere: The Vasculopathic Phenotype

Anti-centromere antibodies (ACA) identify patients with limited cutaneous disease [5,23,24,25,26,27,28,29,30,31,32,33,34,35], prominent vascular dysfunction, and late-onset pulmonary hypertension. Their sera are enriched in anti-endothelial cell reactivity but lack strong profibrotic signaling, consistent with vasculopathic—not fibrotic—endophenotypes. ACA-positive patients have 10–15% lifetime pulmonary arterial hypertension risk but low interstitial lung disease incidence.

#### 4.2.2. Anti-Scl-70: The Fibrotic Archetype

Anti-topoisomerase I (anti-Scl-70) defines fibrotic archetypes [23,24,25,26,27,28,29,30,31,32,33,34,35,52]: diffuse cutaneous involvement, early ILD (present in 40–60% at diagnosis), and TGF-β pathway activation. Single-cell RNA-seq of skin fibroblasts from anti-Scl-70-positive patients reveals transcriptional up-regulation of collagen synthesis, integrins, and myofibroblast differentiation genes—confirming molecularly distinct fibro-inflammatory programs.

#### 4.2.3. Anti-RNA Polymerase III: The Paraneoplastic Cluster

Anti-RNA polymerase III (anti-Pol III) antibodies demarcate a third cluster [49,50,51,52,53]: rapidly progressive skin thickening, scleroderma renal crisis (15–20% incidence vs. 2–5% in other subsets), and temporal association with solid malignancies. POLR3A gene mutations within tumors generate neoantigens triggering cross-reactive immunity, explaining this subset’s paraneoplastic nature. Cancer occurs in approximately 20–25% within 3–5 years of SSc diagnosis in anti-Pol III-positive patients.

#### 4.2.4. Structural Correlates

SSc exemplifies how autoantibody structure influences pathogenesis. IgG subclass distribution varies: ACA are enriched in IgG4 [112,118] (less inflammatory), whereas anti-Scl-70 and anti-Pol III skew toward IgG1/3 (complement-fixing), aligning with clinical aggressiveness.

#### 4.2.5. Clinical Application

Evidence positions SSc as an antibody-defined taxonomy prototype: each autoantibody is an endophenotypic code predicting organ tropism, molecular pathway activation, and therapy response. This underpins precision approaches—early antifibrotic treatment in anti-Scl-70-positive ILD [27,29,30,52], malignancy screening in anti-Pol III disease.

### 4.3. Idiopathic Inflammatory Myopathies: Serological Revolution

IIMs have undergone conceptual revolution through myositis-specific antibody (MSA) and myositis-associated antibody (MAA) discovery. What was once a single clinical category is subdivided into discrete endophenotypes—each with unique molecular drivers and outcome profiles.

#### 4.3.1. Antisynthetase Syndrome

Anti-Jo-1 and other antisynthetase antibodies (anti-PL-7, anti-PL-12, anti-EJ, anti-OJ) [36,37,38,39,163,164,165,166,167,168,169] mark antisynthetase syndrome—a systemic entity combining myositis, ILD (60–90% prevalence), arthritis, Raynaud’s phenomenon, and mechanic’s hands. Antibodies recognize aminoacyl-tRNA synthetases, linking cytoplasmic protein translation to immune activation. Patients display IFN-γ and IL-6-dominated transcriptional signatures in muscle and lung.

#### 4.3.2. Anti-MDA5: The Lethal ILD Phenotype

Anti-MDA5 (melanoma differentiation-associated protein 5) delineates a distinct, often lethal endophenotype [40,41,42,43,44]: rapidly progressive ILD (occurring in 70–90% of anti-MDA5-positive dermatomyositis), cutaneous ulcerations, and vasculopathy. Six-month mortality reaches 40–50% in severe cases without aggressive immunosuppression. Mechanistically, MDA5 is a cytosolic RNA sensor; its targeting implies self-amplifying IFN-I loops mimicking viral responses. Anti-MDA5-positive patients exhibit the highest systemic IFN scores among myositis subsets, validating antibodies as intracellular pathway activation readouts.

#### 4.3.3. Anti-TIF1γ and Anti-Mi-2

Anti-TIF1γ (transcription intermediary factor 1γ) identifies dermatomyositis associated with malignancy [48,163] (cancer prevalence 20–30% within 3 years), while anti-Mi-2 corresponds to more benign, skin-predominant variants [7,36,37] with robust treatment response (>80% improvement with corticosteroids).

#### 4.3.4. Necrotizing Myopathy

Anti-SRP and anti-HMGCR antibodies mark necrotizing autoimmune myopathy, often triggered by statins or viral peptides, characterized by severe muscle weakness with minimal inflammation on biopsy.

#### 4.3.5. Structural Insights

MSAs provide prognostic clarity impossible by histopathology alone. Anti-MDA5 and anti-TIF1γ patients exhibit poor outcomes despite minimal muscle necrosis, whereas anti-Mi-2-positive individuals recover with standard therapy. Elevated afucosylated IgG1 in anti-MDA5 disease correlates with severe ILD, suggesting structural remodeling amplifies effector potential.

#### 4.3.6. Clinical Impact

IIMs epitomize transitions from descriptive nosology to mechanistic classification—each antibody a key to different immunopathogenic corridors. Therapeutic trials increasingly stratify participants by antibody status, acknowledging “myositis” is not a disease but an immunological umbrella.

### 4.4. Primary Sjögren’s Disease: Beyond the Canonical Triad

SjD has long been anchored in the classical anti-Ro52, anti-Ro60, and anti-La/SSB antibody triad. Yet these specificities, once lumped as “SSA/SSB positivity,” are now separated into distinct biological streams.

#### 4.4.1. Anti-Ro60 vs. Anti-Ro52

Anti-Ro60 targets an RNA-binding protein central to cellular RNA quality control. Presence correlates with photosensitivity and systemic manifestations but not necessarily glandular destruction. In contrast, anti-Ro52 (TRIM21) binds a cytosolic ubiquitin ligase involved in protein homeostasis and type-I interferon regulation. Anti-Ro52 positivity predicts extraglandular involvement—particularly ILD, vasculitis, and autoimmune overlap syndromes—and frequently co-occurs with anti-Jo-1 or anti-MDA5, reflecting shared interferon pathways across diseases.

#### 4.4.2. Anti-La and Congenital Heart Block

Anti-La often accompanies anti-Ro60 but contributes to increased congenital heart block risk via transplacental antibody transfer (1–2% risk in anti-Ro/La-positive mothers), demonstrating antibody effects extending beyond hosts.

#### 4.4.3. Novel Antibodies

Beyond the canonical trio, novel autoantibodies reshape pSS taxonomy. Anti-SP1, anti-CA6, and anti-PSP antibodies may precede classical seroconversion, marking early or gland-restricted disease. Anti-Ku and anti-NFAT5 antibodies identify patients with overlapping syndromes and aggressive extraglandular evolution.

#### 4.4.4. Lymphoma Risk

Ectopic germinal centers within salivary glands act as local antibody diversification engines. Patients harboring such structures—especially those with high anti-Ro52 titers—show elevated B-cell lymphoma risk (4–10% lifetime risk vs. <1% in general population), establishing autoantibodies as oncogenic transformation sentinels.

#### 4.4.5. Functional Roles

Antibodies in pSS are not passive markers. Anti-Ro52 can opsonize apoptotic material, activate FcRs, and amplify interferon release—closing inflammatory loops sustaining disease. IgA isotype presence hints at mucosal immune involvement, linking oral microbiome to systemic autoimmunity.

### 4.5. Cross-Disease Convergence

Despite traditional CTD separation, autoantibody networks reveal extensive overlap. Anti-Ro52 appears across Sjögren’s syndrome, myositis, and SSc; anti-Ku bridges myositis and lupus; anti-U1-RNP defines mixed CTD. These shared specificities suggest common upstream triggers—interferon pathways, nucleic acid sensing, or defective apoptotic clearance—manifesting differently according to tissue context and genetic backgrounds.

Systems-level analyses show autoantibody co-expression forms modular clusters transcending diagnostic boundaries. A “TRIM21-interferon” module links pSS, MDA5-myositis, and lupus nephritis, while a “topoisomerase-fibrosis” module unites SSc and radiation-induced fibrotic syndromes. Recognizing these interconnections allows CTD re-classification along mechanistic axes—interferon-driven, fibrotic, vasculitic—rather than historical syndromic divisions.

### 4.6. Clinical Implications

Antibody-defined endophenotypes have profound practical implications:

Diagnosis: Early antibody detection enables preclinical identification of at-risk individuals, as seen with anti-Ro or ACPA years before symptom onset.

Prognosis: Each specificity carries organ-specific predictive weight—anti-MDA5 for ILD, anti-Pol III for renal crisis, anti-Ro52 for lymphoma risk.

Therapeutic Stratification: Antibody profiles increasingly guide biologic selection: interferon blockade in MDA5 disease, anti-BAFF therapy in Ro52-dominant pSS, antifibrotic drugs in Scl-70 ILD.

Monitoring: Quantitative or qualitative shifts in antibody repertoires (glycoform remodeling) serve as dynamic treatment response biomarkers.

**Figure 2:**
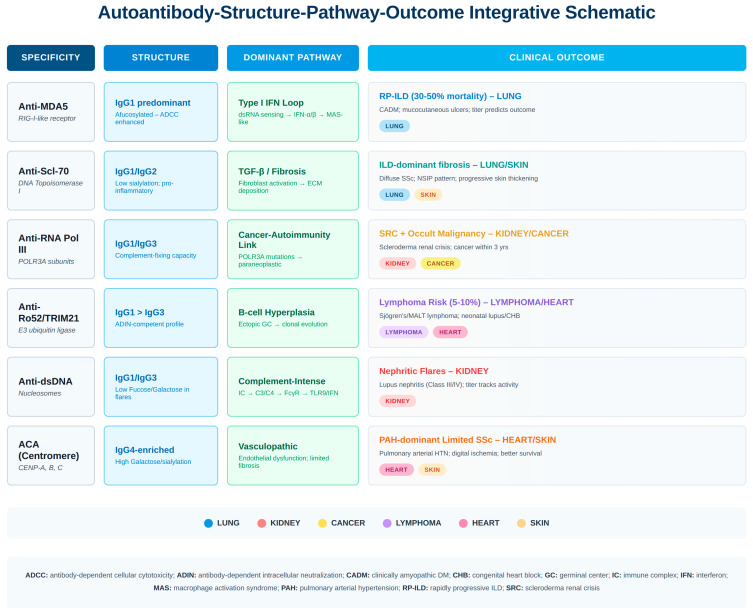
Autoantibody-Structure-Pathway-Outcome Integrative Schematic. This schematic links four layers for six emblematic autoantibodies: (1) Specificity—anti-MDA5, anti-Scl-70, anti-RNA Pol III, anti-Ro52/TRIM21, anti-dsDNA, and ACA (centromere); (2) Structure—typical isotype/subclass and Fc glycosylation bias; (3) Dominant pathway—IFN-I loop, TGF-β/fibrosis, cancer-autoimmunity link, B-cell hyperplasia, complement-intense, and vasculopathic; (4) Clinical outcomes—RP-ILD with 30–50% early mortality, ILD-dominant fibrosis affecting lung and skin, scleroderma renal crisis with occult malignancy, lymphoma risk (5–10%) with cardiac involvement, nephritic flares, and PAH-dominant limited systemic sclerosis. Colored tags indicate primary organ involvement: lung (blue), kidney (pink), cancer (yellow), lymphoma (purple), heart (pink), and skin (orange). Abbreviations: ADCC, antibody-dependent cellular cytotoxicity; ADIN, antibody-dependent intracellular neutralization; CADM, clinically amyopathic dermatomyositis; CHB, congenital heart block; GC, germinal center; IC, immune complex; IFN, interferon; MAS, macrophage activation syndrome; PAH, pulmonary arterial hypertension; RP-ILD, rapidly progressive interstitial lung disease; SRC, scleroderma renal crisis.

Serology evolves from static measurement to functional immunological state readouts—essentially “liquid biopsies” of immune system architecture. Figure 2 synthesizes how selected autoantibody specificities integrate structural features, dominant pathways, and clinical outcomes into distinct endotypes.

## 5. Toward Immuno-Molecular Taxonomy

For more than half a century, CTDs have been defined primarily by clinical syndromes—frameworks built around patient phenotype and symptom constellations rather than the underlying behaviour of the immune system. This approach served earlier eras but now proves insufficient in the face of expanding molecular datasets. Advances in autoantibody profiling, transcriptomics, and proteomics demonstrate that conditions labelled lupus, systemic sclerosis, or SjD are not, as such, singular biological entities but of distinct immune programs converging on overlapping clinical manifestations. The emerging frontier is a redefinition of nosology grounded in molecular language, replacing morphology-based categories with immunological dialects derived from autoantibody networks, gene expression patterns, and cellular ecosystem architecture.

### 5.1. The Endophenotype Framework

The Clinical phenotype captures consequence; endophenotype captures mechanism. An endophenotype is a biologically coherent subset defined by measurable molecular traits—autoantibody profiles [170,171,172], cytokine signatures, transcriptomic modules—mediating between genotype and outward manifestation.

This framework dissolves traditional borders: patients with anti-MDA5 myositis and anti-Ro52 SjD may share dominant IFN-I axes and lung-tropic inflammation despite differing chart diagnoses. Conversely, two “lupus” patients—one IFN-high, one complement-low—represent divergent immunological realities.

Large-scale clustering studies support this new order. Integration of >10,000 SLE transcriptomes reveals recurrent molecular groups—interferon-dominant, plasmablast-rich, neutrophil-NETotic, and metabolic-repressed—each with characteristic antibody repertoires. Similar work in SSc delineates fibrotic-dominant (anti-Scl-70), vasculopathic (ACA), and oncogene-linked (anti-Pol III) clusters—not subtypes invented by statisticians but biological species within CTD genera.

### 5.2. Autoantibodies as Integrative Nodes

Autoantibodies function as highly effective disease classifiers because they integrate three foundational layers of biological information: genetic predisposition, environmental exposure, and immunological context. HLA alleles shape the universe of presentable self-peptides, while polymorphisms in immune-signaling pathways modulate tolerance thresholds; infections, microbiota composition, drugs, and tissue injury determine which antigens are unveiled and when; and the surrounding cytokine milieu, B-cell subset architecture, and metabolic state govern the structure, glycosylation, and effector profile of the resulting antibodies. Together, these forces render autoantibodies condensed molecular biographies—records of where the immune system has been, what it has encountered, and how it has responded. Unlike transient transcriptional programs, autoantibodies persist, storing immunological memory in biochemical form and enabling longitudinal interpretation: the evolution of serological patterns becomes a readable diary of disease trajectory and immune adaptation over time.

### 5.3. Multi-Omic Integration

Practical tasks involve integrating serology with other “-omics” into coherent taxonomy. Prototypical workflows include:

Seromics: High-density antigen arrays identifying hundreds of specificities simultaneously.

Transcriptomics: Defining co-expressed gene modules (IFN, B-cell, fibrosis).

Proteomics and Metabolomics: Quantifying cytokines, complement fragments, metabolic cues.

Spatial and Single-Cell Technologies: Revealing immune interaction geography within tissues [173,174].

When combined, antibody-defined subsets acquire molecular depth. Anti-MDA5 myositis aligns with IFN-I transcriptomic modules and high CXCL10 plasma signatures; anti-Scl-70 sclerosis corresponds to fibroblast TGF-β modules; anti-Pol III disease shows tumor-associated neoantigen expression. Such concordance transforms classification from correlation to causality.

### 5.4. Computational Approaches

Dataset complexity demands computational assistance. Machine learning algorithms already outperform human intuition in recognizing multidimensional disease patterns. Unsupervised clustering rediscovers known antibody subsets and identifies previously unrecognized ones defined by unique sero-transcriptomic signatures. Graph-based network analysis visualizes antibody co-expression as modular constellations—each representing immune pathways.

For example, a “TRIM21-interferon” module spans SjD, MDA5-myositis, and lupus nephritis; a “topoisomerase-fibrosis” module links SSc and radiation-induced fibrotic syndromes. Such networks suggest autoantibody production is organized around conserved immunological hubs rather than being random.

Artificial intelligence further enables predictive modeling: integrating baseline antibody profiles with longitudinal data to forecast flares, organ involvement, or therapeutic response. In clinical trials, AI-assisted seroprofiling could reduce heterogeneity by pre-stratifying participants according to molecular risk, increasing statistical power and decreasing failure rates.

### 5.5. Clinical Applications

Reclassifying CTDs through this lens yields immediate clinical dividends:

Diagnosis: Antibody panels combined with transcriptomic fingerprints allow earlier, more accurate disease trajectory identification.

Prognosis: Structural variants (afucosylated anti-MDA5, IgA anti-Ro) refine risk prediction beyond titer alone.

Disease Monitoring: Dynamic tracking of antibody glycoforms or isotype shifts may serve as non-invasive tissue inflammation surrogates.

Therapeutic Guidance: Specific antibody-defined clusters respond differentially to B-cell depletion, interferon blockade, or antifibrotic therapy.

Antibodies become navigational instruments in precision rheumatology—pointing not only to what patients have but to what will likely happen next.

### 5.6. Rethinking Clinical Trials

Conventional trial design—treating “lupus” or “scleroderma” as homogeneous populations—inevitably dilutes therapeutic signals. Serological taxonomy enables mechanism-anchored trials: testing interferon blockade in anti-MDA5 or anti-Ro52 interferon-high clusters, antifibrotics in anti-Scl-70 fibrotic clusters, or B-cell modulation in plasmablast-rich groups.

Early efforts like PRECISION-SLE and DECODE-SSc consortia exemplify this approach, integrating antibody status and molecular signatures into enrollment criteria. Such designs promise smaller, faster, more interpretable studies, echoing oncology’s transformation from organ-based to mutation-based therapy.

### 5.7. The Mechanism-Based Vision

The future of CTD classification is moving towards unified, mechanism-based categories that combine autoantibody profiles, multi-omic signatures, and clinical phenotypes. Distinct immune “families” are already emerging: interferon-driven diseases (anti-MDA5, anti-Ro52, anti-RNP), fibrotic-matrix disorders (anti–topoisomerase I, anti-Th/To, anti-PM/Scl), vasculopathic or complement-amplified syndromes (anti-C1q, antiphospholipid antibodies), onco-immune clusters (anti–RNA polymerase III, anti-TIF1γ), and B-cell hyperplastic phenotypes (high-titer anti-Ro52 Sjögren’s with lymphoma risk) [175].

These groupings reflect shared immune circuits rather than organ-based coincidences and point toward a future where entities such as “anti-MDA5 disease” or “Scl-70 fibrotic syndrome” function as legitimate diagnostic labels—much like oncology’s shift from anatomical to molecular classification.

The transition is not simple: patients often show multiple antibodies, move between endotypes over time, or develop hybrid profiles. Clinicians will need adaptive taxonomies and new infrastructures—centralized antibody profiling, routine interferon/fibrosis transcriptomics, and integrative dashboards translating complex data into actionable categories.

Eventually, each patient may carry an “immunological passport”—a composite code summarizing their dominant pathways, organ risks, and likely treatment responses. Moving toward this vision demands tight collaboration across rheumatology, immunology, omics, and computational sciences, with autoantibodies as the shared language.

## 6. Autoantibody-Disease Associations: A Comprehensive Framework

The integration of antibody specificity, target organ, and mechanistic insight provides unifying frameworks for understanding CTDs. Table 1 summarizes major antibodies and their associated endophenotypes across diseases.

### 6.1. The Unique Case of Anti-DFS70

Anti-DFS70 antibodies, directed against transcription co-activator LEDGF/p75, occupy paradoxical positions in autoimmunity. They are common in healthy individuals—found in 10–15% of general populations—yet rare in classical systemic autoimmune diseases. When anti-DFS70 occurs in isolation, it carries negative predictive value for systemic disease (>95% specificity for absence of CTD), often representing benign or reactive immune responses.

Clinically, recognizing isolated anti-DFS70 patterns on ANA testing helps avoid over-diagnosis and unnecessary referral. In emerging CTD taxonomy, anti-DFS70 represents the serological counterweight—the reminder that not all autoantibodies signify disease, and immune recognition can occur without immune injury.

### 6.2. ANCA-Associated Vasculitides

ANCA antibodies targeting neutrophil granule enzymes proteinase 3 (PR3) and myeloperoxidase (MPO) extend antibody-defined endophenotype concepts into vasculitis realms. They are not merely diagnostic tools for granulomatosis with polyangiitis (GPA) or microscopic polyangiitis (MPA)—they directly participate in pathogenesis by priming neutrophils, triggering NETosis, complement activation, and endothelial injury.

Interestingly, ANCA biology connects to CTD spectra through overlap syndromes and drug-induced autoimmunity. MPO-ANCA positivity may appear in SLE, SSc, or anti-GBM overlap, often signaling more vasculitic and fibrosing phenotypes. PR3-ANCA associates with granulomatous inflammation and higher relapse rates (50–60% at 5 years vs. 35–40% for MPO-ANCA).

Mechanistically, ANCA responses exemplify how antibodies transmute from epiphenomenal markers to direct effector molecules reshaping vasculature itself.

Selected mechanistic links between autoantibody structure, immune pathways, and clinical outcomes are summarized in Supplementary Table S1.

## 7. Plasmablasts and Antibody-Producing Niches

The final common pathway of humoral autoimmunity is B-cell differentiation into plasmablasts and plasma cells—antibody factories translating immune dysregulation into pathology. In CTDs and ANCA-associated vasculitides (AAV), plasmablasts are not passive bystanders; they are active effectors, immunological sensors, and paradoxically, potential disease activity biomarkers.

### 7.1. Plasmablast Dynamics in Systemic Autoimmunity

Plasmablasts represent short-lived, migratory antibody-secreting cell phases, circulating briefly before homing to tissue niches (bone marrow, inflamed glands, fibrotic lungs). In health, they account for <1% of peripheral B cells; in active systemic autoimmunity, proportions surge to 30–40%, reflecting ongoing extrafollicular B-cell activation.

In SLE, expanded plasmablast pools mirror disease flares and correlate with anti-dsDNA titers, complement consumption, and interferon activity. Transcriptomic profiling reveals shared “plasmablast signatures”—up-regulation of IRF4, BLIMP1, XBP1, and IFN-stimulated genes—across lupus, myositis, and SjD, underscoring convergent humoral pathways.

In Sjögren disease, plasmablast infiltration contributes to the formation of tertiary lymphoid structures (TLS) within salivary glands, enabling local antigen presentation and sustained autoantibody production independent of systemic lymphoid organs. These ectopic germinal centre-like niches recapitulate key organisational features: segregated T/B zones, follicular dendritic cell networks expressing CXCL13 and BAFF, and high endothelial venules supporting lymphocyte recruitment. Within TLS, plasmablasts undergo iterative rounds of affinity maturation, producing high-affinity anti-Ro/SSA and anti-La/SSB antibodies with restricted VH gene usage (VH3-23, VH1-69 over-representation).

Critically, the chronic B-cell stimulation within SjD TLS creates a pre-malignant microenvironment. Prolonged exposure to BAFF, IL-6, and NFκB activation—combined with loss of negative selection checkpoints—drives stepwise accumulation of genetic aberrations (chromosomal translocations involving *BCL2*, *BCL6*, or *MALT1*) culminating in mucosa-associated lymphoid tissue (MALT) lymphoma. Studies using high-throughput BCR sequencing have documented clonal expansions years before overt lymphoma diagnosis, with specific stereotyped BCR sequences (particularly those recognising rheumatoid factor epitopes) conferring higher transformation risk. The ratio of TLS-associated plasmablasts to regulatory T cells, and the extent of PD-1/PD-L1 exhaustion markers, may serve as early biomarkers of lymphomagenesis.

SSc shows fewer circulating plasmablasts but increased bone marrow homing and tissue-resident plasma cells, consistent with “fixed” fibrotic phenotypes rather than relapsing ones.

### 7.2. Plasmablasts in Vasculitis

In AAV, plasmablasts occupy central positions between B-cell activation and effector injury. They produce pathogenic anti-MPO and anti-PR3 antibodies binding to primed neutrophils, triggering NETosis, complement activation, and vascular necrosis.

Interestingly, AAV plasmablasts display highly mutated, antigen-experienced phenotypes—evidence of chronic stimulation rather than naive activation. Their persistence during remission predicts relapse, suggesting plasmablasts function as pathogenic memory reservoirs.

Therapeutically, this explains why rituximab, depleting CD20+ B cells but sparing plasmablasts, induces remission but not complete serological silence. By contrast, proteasome inhibitors (bortezomib) or anti-CD38 therapies (daratumumab) targeting plasmablasts and plasma cells achieve deeper, more durable immunological remission in refractory cases.

### 7.3. Tissue Niches and Survival Signals

Both CTDs and vasculitis rely on specialized survival niches for long-lived antibody-secreting cells. Chemokines (CXCL12), survival factors (BAFF, APRIL), and metabolic cues from stromal cells create microenvironments in bone marrow, salivary glands, and inflamed kidneys protecting plasmablasts from apoptosis.

Interferon-rich milieu—particularly type I and III interferons—amplify survival, forming self-sustaining “plasmablast-interferon loops.” Therapeutically, disrupting these niches through BAFF blockade, CXCR4 antagonism, or metabolic modulation (glutaminase inhibition) represents promising frontiers in restoring immune equilibrium without systemic immunosuppression.

### 7.4. Plasmablasts as Biomarkers

Because of rapid turnover and sensitivity to immune activation, plasmablasts are valuable real-time disease activity biomarkers. Their enumeration by flow cytometry or transcriptomic signatures correlates with flare risk more tightly than static autoantibody titers (area under curve 0.75–0.85 for plasmablast frequency vs. 0.60–0.70 for titers in predicting SLE flares).

Emerging imaging approaches using radiolabeled anti-CD38 tracers may soon allow in vivo visualization of plasmablast-rich niches, providing both diagnostic and prognostic information.

Ultimately, plasmablasts bridge pathogenesis and precision therapy—they are immune system handwriting, recording inflammatory intent in every secreted antibody. Understanding their lifecycle enables clinicians not merely to suppress disease but to edit humoral stories it tells.

## 8. Clinical Relevance: From Endotypes to Anticipatory Medicine

In everyday practice, clinicians diagnose CTDs through clinical constellations—rash and arthritis for lupus, sclerodactyly and Raynaud’s for SSc. This phenotypic approach describes disease appearance but not behaviour. The emerging endotype concept—mechanistic subgroups defined by molecular pathways and autoantibody profiles—provides missing links between immunology and clinical decision-making.

### 8.1. The Endotyping Framework in Practice

Endotyping reframes CTDs as different puzzles of the immune system rather than unitary diseases. Each endotype reflects dominant biological circuits—interferon-driven, fibrotic-matrix, vasculopathic, or B-cell hyperplastic—dictating organ involvement, prognosis, and therapeutic response (Figure 1).

For instance:

Interferon-High Endotype (anti-MDA5, anti-Ro52, anti-RNP): Predicts lung inflammation and robust response to JAK inhibition or interferon blockade.

Fibrotic-TGFβ Endotype (anti-Scl-70, anti-Th/To, anti-PM/Scl): Signals progressive skin and lung fibrosis where anti-IL-6 or antifibrotic therapy is prioritized.

Anti-Pol III Endotype: Anticipates early renal crisis (15–20% risk) and paraneoplastic potential (20–25% cancer risk within 3–5 years), prompting blood pressure vigilance and cancer screening.

Isolated Anti-DFS70 Profile: Often represents benign immunological footprints, safely averting unnecessary immunosuppression.

### 8.2. Anticipatory Medicine

Clinically, endotypes enable anticipatory medicine—tailoring monitoring and treatment intensity to biological risk. They clarify heterogeneity in therapeutic trials, identify responders prospectively, and spare low-risk patients from overtreatment.

By integrating serology, transcriptomics, and imaging, clinicians track endotype shifts over time, adjusting therapy dynamically as immune landscapes evolve. For example, a patient with anti-Ro52 antibodies showing IgG1-dominant, hypogalactosylated patterns may be prioritized for aggressive treatment, while IgG4-dominant, sialylated patterns might allow watchful waiting.

### 8.3. Paradigm Shift

**Figure 3:**
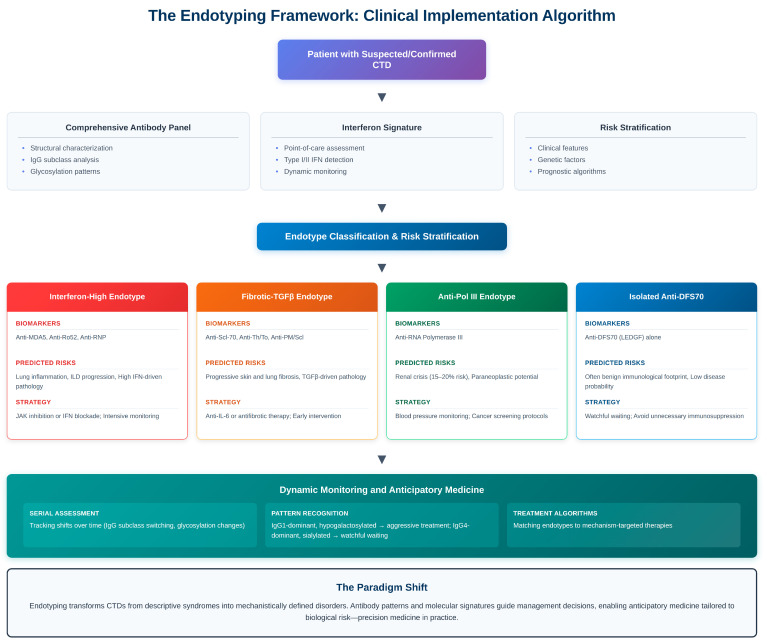
The Endotyping Framework: Clinical Implementation Algorithm for Connective Tissue Diseases. This algorithm depicts the systematic approach to implementing endotype-based precision medicine in clinical practice for connective tissue diseases (CTDs). Abbreviations: CTD, connective tissue disease; IFN, interferon; ILD, interstitial lung disease; IgG, immunoglobulin G; JAK, Janus kinase; IL-6, interleukin-6; TGFβ, transforming growth factor beta; DFS70, dense fine speckled 70; LEDGF, lens epithelium-derived growth factor.

Autoantibodies have evolved from passive diagnostic markers to core mechanistic determinants that define the biology, trajectory, and treatment of connective tissue diseases. They emerge years before symptoms, opening a preclinical window where prediction and early intervention become possible. Serological endotypes now refine diagnosis more accurately than organ-based taxonomies and guide anticipatory monitoring—anti-Scl-70 signalling high ILD risk, anti–RNA polymerase III pointing to renal crisis and malignancy, high-titer anti-Ro52 identifying lymphoma-prone Sjögren disease. Therapeutic choices increasingly follow immune circuits rather than disease names: interferon-driven signatures respond to IFN blockade, anti-MDA5 dermatomyositis requires early combination therapy, anti-HMGCR myopathy relies on IVIG. Dynamic shifts in antibody quality—subclass patterns, glycosylation, new specificities—predict flare or remission earlier than clinical symptoms. Multiplex serological platforms and AI-driven dashboards integrate these profiles with clinical and omic data, clustering patients into mechanistic endotypes and forecasting outcomes. Endotyping is becoming the grammar of CTDs, transforming rheumatology into a discipline of precision immunology grounded in real-time immune signatures (Figure 3).

### 8.4. Clinical Case Vignettes—Endophenotype-Driven Management

Section 8.4 demonstrates how autoantibody-defined endophenotypes radically alter prognosis, monitoring, and treatment timing across connective tissue diseases. The core message is simple and uncomfortable: treating by syndrome alone is often too slow and sometimes lethal (the clinical vignette is available in Appendix A).

Across five archetypal cases (anti-MDA5 DM-ILD, anti-RNA pol III SSc, anti-Ro52 UCTD, anti-Ro52/RF Sjögren’s, anti-HMGCR necrotizing myopathy) of antibody profiles, the following steps are given:

- Identify hidden high-risk biology (e.g., “mild” myositis masking fatal ILD).
- Trigger anticipatory surveillance (renal crisis, cancer, lymphoma) before symptoms appear.
- Dictate mechanism-based therapy (IVIG over steroids in anti-HMGCR; upfront triple therapy in anti-MDA5).
- Prevent therapeutic futility and iatrogenic harm by avoiding ineffective default strategies.

The unifying insight: autoantibodies are not diagnostic ornaments; they are operational risk signals. Acting early on these signals converts catastrophic trajectories (fibrosis, renal failure, disseminated cancer) into controllable disease courses with preserved function and survival.

### 8.5. Diagnostic Algorithms—Autoantibody-Guided Decision Trees

This section translates immunological insights into practical, stepwise diagnostic algorithms, redefining serology as a dynamic clinical decision-support tool rather than a binary diagnostic test (full algorithms are provided in Appendix B).

The Core principles are:

- ANA pattern interpretation combined with clinical context enables targeted reflex testing, avoiding indiscriminate antibody panels.
- Specific autoantibodies define prognostic endophenotypes, each associated with distinct risks that determine surveillance intensity, therapeutic urgency, and monitoring frequency.
- Negative serology does not terminate diagnostic reasoning; longitudinal evolution of antibodies and clinical features remains central to disease recognition.

These algorithms address five major clinical scenarios:

1. Evaluation of a positive ANA, using pattern-driven branching to guide diagnosis toward SLE, systemic sclerosis, idiopathic inflammatory myopathies, or overlap syndromes.
2. Risk stratification in undifferentiated connective tissue disease (UCTD), enabling individualized follow-up schedules and early preventive intervention.
3. Assessment of CTD-associated interstitial lung disease, distinguishing rheumatologic emergencies (anti-MDA5–associated RP-ILD) from more indolent fibrotic pathways (anti-Scl-70).
4. Serological endophenotyping in SLE (renal-dominant, interferon-driven, antiphospholipid-associated, neuropsychiatric), aligning immune biology with contemporary targeted therapies.
5. Rational use of serial autoantibody testing, applied selectively to anticipate disease flares, phenotypic transitions, or malignant transformation—never as routine, low-value testing.

To enhance clinical usability, Table 2 synthesizes serological patterns, prognostic stratification, and management urgency into a unified decision-support framework.

## 9. Therapeutic Horizons: From Suppression to Recalibration

Autoantibodies have moved from passive diagnostic markers to true biological determinants that shape detection, prognosis, and treatment in connective tissue diseases. They emerge years before symptoms—anti-Ro, anti-MDA5, anti-Scl-70—offering a preclinical window where surveillance or early immune-tolerance strategies could prevent irreversible organ damage. This shift mirrors oncology’s transition from late diagnosis to early interception [11,13,120,121,122,123,124,125,126].

Therapeutically, serology predicts response more reliably than clinical labels: interferon-related signatures respond to JAK or IFN-blockade; anti-Scl-70 aligns with antifibrotics and mycophenolate; anti-MDA5 or anti-SRP myositis requires combination regimens rather than steroids alone. Antibodies reveal which immune circuit is active—treatment follows mechanism rather than disease name [102,103,104,105,106,107,108,109,110,111,112,113,114,115,116,117,118,119].

B-cell–directed therapies are becoming more precise: next-generation anti-CD20 antibodies, BAFF and BAFF-R inhibitors, CD38-targeting strategies, and plasma-cell metabolic modulation reshape the autoantibody landscape rather than merely suppress it. FcRn inhibitors, complement blockers, and IVIG derivatives target antibody effector pathways directly, proving that managing consequences can be as effective as reducing production [86,87,88,89,90,91,92,93,94,95,96,97,98,99,100,101,127,128,129,130,131,132,133,134,135,136,137,138,139,140,141,142,143,144,145,146].

However, the translation of autoantibody-guided strategies into routine therapeutic decision-making remains constrained by current assay limitations. The variability between platforms, differences in antigen preparation, limited standardization of cut-offs, and restricted sensitivity to qualitative antibody features (e.g., affinity, glycosylation, or epitope spreading) may obscure biologically relevant signals. IgG glycosylation profiling by mass spectrometry shows inter-laboratory coefficients of variation of 15–25% for minor glycoforms, and ELISA-based autoantibody quantification lacks standardization across manufacturers. These technical constraints highlight the need for harmonized, high-resolution assays to fully exploit autoantibodies as dynamic therapeutic biomarkers rather than static categorical variables. These limitations must temper enthusiasm for biomarker-guided management until robust, standardized assays are validated in prospective clinical trials.

The future lies in restoring tolerance—selective antigen tolerization, CAR-T approaches repurposed for autoimmunity, and plasma-cell reprogramming—all aiming for durable, drug-free remission. Dynamic monitoring of antibody quality (isotype, glycosylation, new specificities) will replace crude titer evaluation, turning serology into a real-time immune activity gauge [147,148,149,150,151,152,153,154,155,156] (Table S1).

Ultimately, auto-antibodies act as stable molecular signatures of each patient’s immune history. They anchor a new CTD taxonomy defined by mechanisms, not symptoms. Precision immunology replaces blanket immunosuppression, integrating serology with multi-omics and imaging into adaptive, individualized care. Autoantibodies—long peripheral—have become the language linking mechanism, phenotype, and prognosis [11,120,121,122,123,124,125,126,157,158,159,160,161,162,163,164,165,166,167,168,169,170,171,172,173,174,175,176,177,178,179].

## Figures and Tables

**Table 1:** Major Autoantibody-Disease Associations and Endophenotypic Features.

Autoantibody	Primary Disease(s)	Organ Tropism	Mechanistic Features	Clinical Implications	References
Anti-dsDNA	SLE	Kidney, skin	IgG1/3-mediated complement fixation; IFN signature	Nephritis risk; disease activity marker	[3,15,16,17]
Anti-C1q	SLE	Kidney	Amplifies complement activation	Renal flare prediction (HR 2.5–3.5)	[18,19,20]
Antiphospholipid (aPL)	SLE, APS	Vascular, placental	Platelet activation; NETosis	Thrombosis; pregnancy loss	[21,22,23]
Anti-Sm	SLE	Systemic	IFN-driven; anti-snRNP complex	High specificity (98%) for SLE	[24,25]
Anti-Ro60 (SSA)	SjD, SLE, SCLE	Skin, heart (fetal)	RNA quality control disruption	Photosensitivity; CHB risk (1–2%)	[26,27,28]
Anti-Ro52 (TRIM21)	Sjögren’s, myositis, SSc	Lung, systemic	IFN regulation; ubiquitin ligase	Extraglandular disease; lymphoma risk	[29,30,31]
Anti-La (SSB)	SjD, SLE	Salivary glands	Often co-occurs with anti-Ro	CHB risk; glandular disease	[32,33]
Anti-centromere (ACA)	SSc (limited)	Vasculature, lung	IgG4-dominant; vasculopathic	PAH risk (10–15%); low ILD risk	[34,35,36]
Anti-Scl-70 (topo I)	SSc (diffuse)	Skin, lung	IgG1/3; TGF-β activation	ILD (40–60%); skin fibrosis	[37,38,39]
Anti-RNA pol III	SSc (diffuse)	Kidney, skin	Paraneoplastic; renal crisis	SRC risk (15–20%); cancer (20–25% in 3–5 y)	[40,41,42]
Anti-U3-RNP (fibrillarin)	SSc	Heart, lung	Nucleolar target	Severe organ involvement; poor prognosis	[43,44]
Anti-Th/To	SSc (limited)	Lung	Nucleolar; fibrotic	ILD; limited skin involvement	[45,46]
Anti-PM/Scl	SSc-myositis overlap	Muscle, lung	Nucleolar/exosome complex	Overlap syndrome; better prognosis	[47,48]
Anti-Jo-1	Antisynthetase syndrome	Lung, muscle, joints	Histidyl-tRNA synthetase; IFN-γ	ILD (60–90%); arthritis; mechanic’s hands	[49,50,51]
Anti-PL-7, PL-12	Antisynthetase syndrome	Lung > muscle	Threonyl/alanyl-tRNA synthetase	ILD-predominant	[52,53]
Anti-MDA5	Dermatomyositis	Lung, skin	Cytosolic RNA sensor; IFN-I loop	RP-ILD (70–90%); high mortality (40–50% at 6 mo if severe)	[54,55,56]
Anti-TIF1γ	Dermatomyositis	Skin > muscle	Transcription regulator	Cancer-associated (20–30% in 3 y)	[48]
Anti-Mi-2	Dermatomyositis	Skin, muscle	Chromatin remodeling	Good prognosis; steroid-responsive (>80%)	[60,61]
Anti-SRP	Necrotizing myopathy	Muscle	Signal recognition particle	Severe weakness; statin-associated	[62,63]
Anti-HMGCR	Necrotizing myopathy	Muscle	HMG-CoA reductase	Statin-triggered; severe	[64,65,66]
Anti-NXP2	Juvenile dermatomyositis	Muscle, skin	Nuclear matrix protein	Calcinosis; cancer in adults	[67,68]
Anti-SAE	Dermatomyositis	Skin	SUMO-activating enzyme	Dysphagia; skin-predominant	[69,70]
Anti-U1-RNP	MCTD, SLE	Systemic	spliceosome component	Overlap features; Raynaud’s	[71,72]
Anti-Ku	Myositis-SSc overlap	Muscle, lung	DNA repair complex	Overlap syndrome	[73,74]
Anti-DFS70	Healthy individuals	None	LEDGF/p75; protective	Negative predictive value for systemic disease	[75,76]
Anti-PR3 (c-ANCA)	GPA	Vasculature, lung, kidney	Neutrophil priming; NETosis	Granulomatous inflammation; relapse risk	[77,78,79]
Anti-MPO (p-ANCA)	MPA, EGPA	Vasculature, kidney	Neutrophil activation; complement	Vasculitis; glomerulonephritis	[80,81,82]

Abbreviations: APS, antiphospholipid syndrome; CHB, congenital heart block; EGPA, eosinophilic granulomatosis with polyangiitis; GPA, granulomatosis with polyangiitis; HR, hazard ratio; ILD, interstitial lung disease; MCTD, mixed connective tissue disease; MPA, microscopic polyangiitis; PAH, pulmonary arterial hypertension; RP-ILD, rapidly progressive interstitial lung disease; SCLE, subacute cutaneous lupus erythematosus; SRC, scleroderma renal crisis.

**Table 2:** Autoantibody-Defined Endotypes: Immune Pathways, Organ Risks, and Clinical Decision-Making Framework.

Autoantibody	Primary Disease Context	Dominant Immune Pathway	Key Organ Risk	Clinical Implications
Anti-dsDNA	SLE	Complement activation, immune complex deposition	Kidney	Lupus nephritis risk; disease activity monitoring
Anti-Sm	SLE	IFN-driven autoimmunity	Systemic	High specificity for SLE; severe disease phenotype
Anti-Ro60 (SSA)	Sjögren’s, SLE, SCLE	RNA quality control disruption	Skin, fetal heart	Photosensitivity; congenital heart block risk
Anti-Ro52 (TRIM21)	Sjögren’s, myositis, SSc	Type I interferon amplification	Lung, lymphoid tissue	ILD risk; lymphoma surveillance; treatment stratification
Anti-centromere	Limited SSc	Vasculopathy-dominant	Pulmonary vasculature	PAH risk; relatively low ILD risk
Anti-Scl-70 (topo I)	Diffuse SSc	Fibrotic signaling (TGF-β)	Lung, skin	Progressive ILD; antifibrotic consideration
Anti–RNA polymerase III	Diffuse SSc	Paraneoplastic/vascular stress	Kidney, systemic	Scleroderma renal crisis; malignancy surveillance
Anti-MDA5	Dermatomyositis	IFN-I loop, antiviral response	Lung	Rapidly progressive ILD; rheumatologic emergency
Anti-TIF1γ	Dermatomyositis	Tumor-associated immune response	Systemic	Cancer-associated myositis; intensive screening
Anti-Jo-1	Antisynthetase syndrome	IFN-γ–skewed inflammation	Lung, muscle	ILD-predominant disease; immunosuppression escalation
Anti-PL-7/Anti-PL-12	Antisynthetase syndrome	tRNA synthetase–linked immunity	Lung	Severe ILD; poorer muscle involvement
Anti-HMGCR	Necrotizing myopathy	Antibody-mediated myotoxicity	Muscle	IVIG-first strategy; steroid resistance
Anti-SRP	Necrotizing myopathy	Cytotoxic muscle injury	Muscle, bulbar	Severe weakness; aggressive therapy required
Anti-DFS70	Healthy/ANA+ individuals	Non-pathogenic/protective	None	Negative predictive value for systemic CTD

## Data Availability

No new data were created or analyzed in this study. Data sharing is not applicable to this article.

## Supplementary Materials

The following supporting information can be downloaded at: https://www.mdpi.com/article/10.3390/antib15010007/s1, Table S1: Integrative Summary: Autoantibody Specificity, Structure, Pathways, and Outcomes.

## Abbreviations

AAV	ANCA-associated vasculitis
ACE	Angiotensin-converting enzyme
ADCC	Antibody-dependent cellular cytotoxicity
ANA	Antinuclear antibody
ANCA	Anti-neutrophil cytoplasmic antibody
aPL	Antiphospholipid antibodies
APS	Antiphospholipid syndrome
APRIL	A Proliferation-Inducing Ligand
BAFF	B-cell Activating Factor
CHB	Congenital heart block
CK	Creatine kinase
CTD	Connective tissue disease
DC-SIGN	Dendritic Cell-Specific Intercellular adhesion molecule-3-Grabbing Non-integrin
DFS70	Dense fine speckled 70
DLCO	Diffusing capacity of the lungs for carbon monoxide
EGPA	Eosinophilic granulomatosis with polyangiitis
ENA	Extractable nuclear antigens
Fc	Fragment crystallizable (region of immunoglobulin)
FcRn	Neonatal Fc receptor
FcγR	Fc gamma receptor
FVC	Forced vital capacity
GlcNAc	N-acetylglucosamine
GPA	Granulomatosis with polyangiitis
HLA	Human leukocyte antigen
HMGCR	3-hydroxy-3-methylglutaryl-coenzyme A reductase
HR	Hazard ratio
HRCT	High-resolution computed tomography
IFN	Interferon
IgG	Immunoglobulin G
ILD	Interstitial lung disease
IPAF	Interstitial pneumonia with autoimmune features
IVIG	Intravenous immunoglobulin
JAK	Janus kinase
LEDGF	Lens epithelium-derived growth factor
LN	Lupus nephritis
MAA	Myositis-associated antibodies
MALT	Mucosa-associated lymphoid tissue
MCTD	Mixed connective tissue disease
MDA5	Melanoma differentiation-associated protein 5
MPA	Microscopic polyangiitis
MPO	Myeloperoxidase
MSA	Myositis-specific antibodies
NETosis	Neutrophil extracellular trap formation
NSIP	Nonspecific interstitial pneumonia
PAH	Pulmonary arterial hypertension
pDC	Plasmacytoid dendritic cell
PFT	Pulmonary function test
PR3	Proteinase 3
pSS	Primary Sjögren syndrome
RF	Rheumatoid factor
RP-ILD	Rapidly progressive interstitial lung disease
SCLE	Subacute cutaneous lupus erythematosus
SjD	Sjögren disease
SLE	Systemic lupus erythematosus
SRC	Scleroderma renal crisis
SRP	Signal recognition particle
SSc	Systemic sclerosis
TGF-β	Transforming growth factor beta
TIF1γ	Transcription intermediary factor 1 gamma
TLR	Toll-like receptor
TLS	Tertiary lymphoid structures
TRIM21	Tripartite motif-containing protein 21
UCTD	Undifferentiated connective tissue disease
UIP	Usual interstitial pneumonia

## Appendix A. Clinical Case Vignettes

Cases illustrating autoantibody-defined endophenotype-driven management.

Case 1: Anti-MDA5 DM with RP-ILD

42yo woman: mechanic’s hands, dyspnea, CK 380, HRCT ground-glass 30%, FVC 68%, DLCO 52%, anti-MDA5+. Management: Triple therapy 48 h (methylpred 1 g IV ×3, tacrolimus, IVIG 2 g/kg). Outcome: FVC 72% at 6 mo.

Case 2: SSc with Anti-RNA pol III

58yo man: rapid skin progression (mRSS 28), anti-RNA pol III+. Management: SRC surveillance (twice-daily BP) + malignancy screening (CT). Outcome: Lung adenocarcinoma detected at 8 mo—curative resection.

Case 3: UCTD with Anti-Ro52 to SLE

32yo woman: arthralgias, photosensitivity, ANA 1:640, anti-Ro52 > 200. Management: q3 mo monitoring. Outcome: Class III + V nephritis at 18 mo—complete response with MMF + belimumab.

Case 4: Sjogren’s Lymphoma Risk

48yo woman: 5 yr pSS, hypergammaglobulinemia, RF 180, anti-Ro52 185. Management: Salivary US q6–12 mo. Outcome: MALT lymphoma IE at 4 yr—localized radiation, complete remission.

Case 5: Anti-HMGCR Myopathy

55yo man: statin 8 yr, proximal weakness, CK 8500, anti-HMGCR+. Management: IVIG first-line (not high-dose steroids). Outcome: CK normalized 3 mo, near-normal function at 3 yr on IVIG.

## Appendix B. Diagnostic Algorithms

**Table A1:** ANA Pattern Evaluation.

Pattern	Antibody Panel	Interpretation
Homogeneous	dsDNA, Sm, RNP, Ro/La, C3/C4	dsDNA + Sm + low C = SLE
Nucleolar	Scl-70, RNA pol III, PM/Scl	Scl-70 = ILD; RNA pol III = SRC + cancer
Speckled + weakness	Jo-1, MDA5, TIF1g, HMGCR	MDA5 = RP-ILD EMERGENCY
Centromere	CENP-A/B, AMA	lcSSc; screen PAH

**Table A2:** UCTD Risk.

LOW	Isolated ANA 1:160–320; DFS70+	HCQ; annual follow-up
MODERATE	ANA > 1:320; Ro/La+; RNP+	HCQ mandatory; q3-6 mo; 30–40% progression
HIGH	dsDNA+; Sm+; low C; Ro52 > 200	q3 mo intensive monitoring; >60% to SLE

**Table A3:** CTD-ILD.

Antibody	Pattern	Management
Anti-Scl-70	NSIP/UIP	MMF or CYC; nintedanib if progressive
Anti-MDA5	OP/DAD	EMERGENCY: Triple therapy within 48 h
Anti-Jo-1	NSIP/OP	Pred + MMF; rituximab if refractory

ASS = antisynthetase; AZA = azathioprine; CYC = cyclophosphamide; DAD = diffuse alveolar damage; HCQ = hydroxychloroquine; MMF = mycophenolate; OP = organizing pneumonia; RP-ILD = rapidly progressive ILD; RTX = rituximab.
