# Supplementary material for: Autoantibodies as Precision Tools in Connective Tissue Diseases: From Epiphenomenon to Endophenotype
Source: Antibodies (Basel). 2026 Jan 13;15(1):7. doi: 10.3390/antib15010007 (PMC12821479; doi:10.3390/antib15010007)
Supplement: Supplementary file 1 [file antibodies-15-00007-s001.zip › antibodies-4033004-supplementary.pdf]

Table S1. Integrative Summary: Autoantibody Specificity, Structure, Pathways, and Outcomes.

Autoantibody	Disease	Structure	Pathway	Clinical Outcome
Anti-dsDNA	SLE	IgG1/3; hypogalactosylated	Complement; IFN-I	Nephritis; activity marker
Anti-MDA5	DM	IgG1; afucosylated	IFN-I amplification	RP-ILD; high mortality
Anti-Scl-70	dcSSc	IgG1/3; pro-inflammatory	TGF-beta fibrosis	ILD; skin fibrosis
Anti-RNA pol III	dcSSc	Paraneoplastic	Tumor cross-reactivity	SRC; cancer risk
Anti-centromere	lcSSc	IgG4; sialylated	Vasculopathic	PAH; low ILD
Anti-Ro52	SjD, overlap	IgG1 predominant	IFN regulation	Extraglandular; lymphoma
Anti-Jo-1	ASS	IgG1/2	IFN-gamma; Th1	ILD; arthritis
Anti-HMGCR	IMNM	Complement-fixing	Complement myotoxicity	Necrotizing myopathy

ASS=antisynthetase; dcSSc=diffuse cutaneous SSc; DM=dermatomyositis; IMNM=immune-mediated necrotizing myopathy; lcSSc=limited cutaneous SSc; PAH=pulmonary arterial hypertension; RP-ILD=rapidly progressive ILD; SjD=Sjogren; SLE=systemic lupus; SRC=scleroderma renal crisis.

This supplementary table provides a mechanistic integration of selected autoantibodies across connective tissue diseases, linking antibody specificity to immunoglobulin structural features (including subclass and Fc glycosylation patterns), dominant pathogenic pathways, and major clinical outcomes. By connecting molecular characteristics of autoantibodies with downstream immune activation programs and organ-specific manifestations, the table illustrates how serology functions as a stable readout of disease endophenotypes and pathogenic trajectories beyond conventional diagnostic classification.
